# Emergence of Novel *Chlamydia trachomatis* Sequence Types among Chlamydia Patients in the Republic of Belarus

**DOI:** 10.3390/microorganisms10020478

**Published:** 2022-02-21

**Authors:** Valentina A. Feodorova, Yury V. Saltykov, Anna A. Kolosova, Liudmila V. Rubanik, Nikolay N. Poleshchuk, Vladimir L. Motin

**Affiliations:** 1 Federal Research Center for Virology and Microbiology, Branch in Saratov, 410028 Saratov, Russia; saltykov3443@mail.ru (Y.V.S.); koloanyuta@yandex.ru (A.A.K.); 2 Republican Research and Practical Center for Epidemiology and Microbiology, 220114 Minsk, Belarus; rubaniklv@tut.by (L.V.R.); pnn@belriem.by (N.N.P.); 3 Department of Pathology, University of Texas Medical Branch, Galveston, TX 77555, USA; vlmotin@utmb.edu

**Keywords:** *Chlamydia trachomatis*, MLST, chlamydial infection, sequence type, ST, typing, housekeeping genes

## Abstract

*Chlamydia trachomatis* (CT) is a major cause of sexually transmitted diseases worldwide. The multilocus sequence typing (MLST) of clinical samples from random heterosexual chlamydia patients who were either asymptomatic or reported clinical manifestations of genital chlamydiosis (*n* = 63) in each of the seven major regions of the Republic of Belarus in 2017–2018 revealed 12 different CT sequence types (STs). We found seven known STs, ST4, ST6, ST9, ST13, ST38, ST95 and ST110, and five novel variants, namely ST271–ST275, which have not been detected elsewhere thus far. The ST4 variant was predominant (27/63, 42.9%) and detected in six out of seven regions. The two most common STs, ST9 and ST13, were regularly seen in four out of seven regions. In contrast, the remaining STs, ST6, ST38, ST95, ST110, and novel STs271-275, surfaced randomly in different parts of the country. The emergence of novel STs was registered in two regions, namely Minsk (ST271 and ST275) and Brest (ST271, ST272, ST273, and ST274). All the STs of detected CT strains were clustered into two Groups, I and III, which are characteristic of CT urogenital strains. No STs typical for Group II, specific to the LGV strains, were revealed. Our study contributes to better understanding the genetic diversity and molecular evolution of CT, one of the most important pathogens in public health worldwide.

## 1. Introduction

Urogenital chlamydia, caused by *Chlamydia trachomatis* (CT), is one of the most common curable sexually transmitted infections (STIs) worldwide [1]. Chlamydia is considered by the World Health Organization (WHO) to be a global public health burden due to: (i) the high level of prevalence and incidence of the infection with millions of new cases annually [1,2]; (ii) serious complications in the human reproductive system including both male and female infertility in untreated cases; (iii) increased risk of HIV transmission and acquisition [1,2]; (iv) the mild or even asymptomatic course of disease which often prompts the spread of chlamydia infection [3,4]; and (v) the fact that primary infection appears in young individuals, the most sexually active group of humans. According to the global health sector strategy on STIs, generated by the World Health Assembly in 2016, it is critical to expand data collection efforts at the country level to assess chlamydia prevalence rates and incidence to significantly reduce the chlamydia burden worldwide [5].

*M*ulti*l*ocus *S*equence *T*yping (MLST) technique is a molecular typing method that is quite useful for the global epidemiology of bacterial pathogens, including CT isolates [3,4,6]. MLST allows CT strains to be quickly discriminated following rapid and accurate recognition of the emergence of both novel and already-known sequence types (STs) of the pathogen that is critical for epidemiological surveillance, monitoring therapy, and outbreak control over CT-associated infections [6,7,8,9,10].

Comparative evaluation of different typing schemes for CT isolates based on other targets, such as *ompA*, variable number tandem repeat (VNTR) genome loci and multiple loci variable number of tandem repeats (MLVA), multilocus typing (MLT) DNA microarray, and spatial Laser Speckle Contrast Analysis (*s-LASCA* imaging) of virtual gene-based speckles (GB-speckles), proved the MLST as the method of choice for global epidemiological purposes, especially for CT intraspecific discrimination [8,11,12,13,14,15,16]. Recently, for CT, MLST-based phylogenetic trees were found to be similar to whole-genome-based trees as both types of trees showed comparable levels of incongruence in the phylogeny for this bacterial species [7,10,17]. MLST was reported to be more accurate, easy to conduct, had lower costs, and was faster by an order of magnitude compared to its contemporary, CT genome-based ST detection tools [6,10]. More recently, MLST was concluded to be more suitable for intraspecific differentiation of CT than typing based on whole-genome phylogenies, in which the high branch supports could be an artifact probably caused by data size [7].

**Figure 1:**
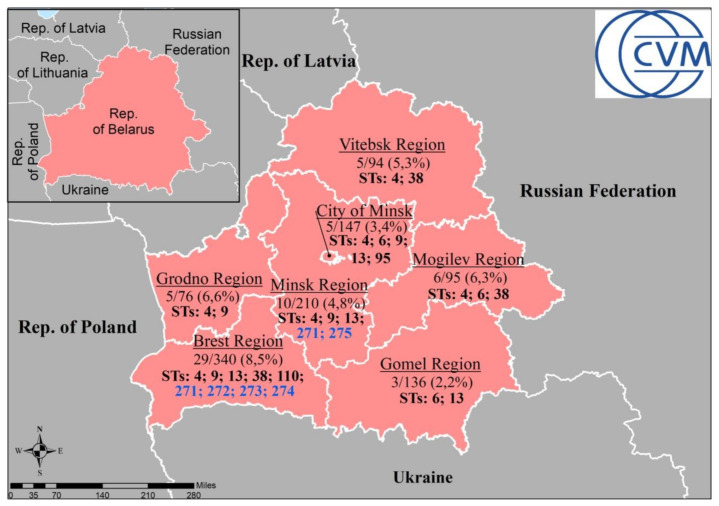
Geographical distributions of *C. trachomatis* STs in the Republic of Belarus among CT-positive patients/totally examined (%). The map was generated with Esri ArcGis Desktop 10.6.1 (www.esri.com (accessed on 10 February 2022)). Novel STs identified in this study are marked in blue.

Several MLST schemes have been developed for CT which are based on PCR amplification and DNA sequencing of five to seven chlamydial genomic loci [3,6,8,9,12,13,14,17]. Two schemes are based on housekeeping genes [3,18] and the third basic system relies on five highly variable genomic loci of non-housekeeping genes such as *hctB* [CT046], CT058, CT144, CT172, and *pbpB* [CT682] [8]. The relevant collections of CT MLST sequences, allele profiles and STs detected globally are available in the open access databases, such as PubMLST database (https://pubmlst.org/organisms/chlamydiales-spp/ (accessed on 21 January 2022)) and the Uppsala University *Chlamydia trachomatis* MLST database (http://mlstdb.bmc.uu.se (accessed on 21 January 2022)). Comparative evaluation of different MLST schemes revealed the Uppsala system as the best high-resolution typing method with a great discriminatory capacity applicable for short-term clinical epidemiology and outbreak investigations of CT [8]. In fact, this system has been successfully applied for identification and differentiation of CT strains related to: (i) the novel “Swedish” variant of CT, nvCT [19,20]; (ii) the predominating genotype E [20,21]; (iii) strains that infected men who have sex with men (MSM) and heterosexuals [17,22,23]; (iv) investigation of the clonal spread of Lymphogranuloma venereum (LGV) strains [24,25]; (v) the role of tissue tropism [22,26]; and (vi) molecular epidemiology and antibiotic treatment of trachoma [17,27,28]. At the same time, the MLST scheme developed by Pannekoek et al. [3] based on seven molecular targets has been recognized as the best possible choice for global epidemiological purposes [11] and monitoring of molecular evolution in CT [14,17,22]. Recently, this system was successfully used to study the distribution and prevalence of the most common CT STs and identification of the novel STs in random multiethnic heterosexual patients, including asymptomatic individuals and couples, who were infected with either wild type of CT or nvCT [4,29,30]. Importantly, this MLST resulted in CT division into three ST groups, Groups I, II and III [3,4,11], with subsequent separation of the urogenital CT strains into Groups I and III, distinguished from the LGV strains forming Group II [3] identified in some countries including Eastern Europe [24,25].The main goal of this study was to investigate the prevalence of CT STs in the Republic of Belarus, which is located in Eastern Europe bordering several countries, such as Poland, Lithuania, Latvia, Russian Federation, and Ukraine (Figure 1) (https://en.wikipedia.org/wiki/Belarus). According to the official statistics [31], the incidence of chlamydia in 2018 in the Republic of Belarus was 51.7 cases per 100,000 population.

## 2. Materials and Methods

Clinical samples (either cervical or urethral swabs) were collected from a random cohort of heterosexual patients (*n* = 1098, women (*n* = 847) and men (*n* = 251)), 18–42 years of age, who visited different regional clinics of the Republic of Belarus in 2017–2018. The specimens were delivered to the diagnostic laboratory of the Republican Research and Practical Center for Epidemiology and Microbiology, Minsk, the Republic of Belarus, in order to be tested for the presence of *C. trachomatis* DNA by PCR as described [4] to confirm ongoing chlamydia infection. The female patients either were asymptomatic or reported clinical manifestations of cervicitis, pelvic inflammatory disease such as pronounced vaginal discharge, inter-menstrual and post-coital bleeding, lower abdominal pain, dysuria, etc., under annual planned clinical examination. Male patients demonstrated mainly symptoms of either urethritis or epididymitis namely of dysuria, urethral discharge, palpable swelling of the epididymis, and a fever in a single case. Each sample was randomly numbered to protect patients’ personal information. This screening was performed within the framework of the National Research and Technical Program “New Methods of Medical Aid Delivery”, SubProgram “Infections and Biological Safety”, 2016–2020. Total DNA was isolated from clinical specimens of suspected chlamydia patients using the DNeasy Blood & Tissue Qiagen Kit (Qiagen, Hilden, Germany). The DNA concentration for each individual specimen was measured with a spectrophotometer (BioRad Laboratories, Redmond, WA, USA) according to the manufacturer’s instructions. DNA samples were routinely analyzed by conventional real-time PCR kits ‘AmpliSens^®^ Chlamydia trachomatis-FRT PCR kits’ (Central Research Institute of Epidemiology, Moscow, Russia) as described previously [4]. All PCR-positive DNA specimens were subjected to PCR amplification of the seven housekeeping genes (*gatA*, *oppA*, *hflX*, *gidA*, *enoA*, *hemN* and *fumC*) for MLST analysis according to Pannekoek et al. [3]. All representative sequences reported in this research were deposited in PubMLST database (https://pubmlst.org/ (accessed on 21 January 2022), Acc. No. 4464–4526). The evolutionary tree was inferred in MEGA 7.0 [32] using the Neighbor-Joining method with 100 bootstrap replicate samples. ModelTest in MEGA 7.0 was used to identify the most appropriate model of evolution (the Tamura 3-parameter method [32]). MLST and GrapeTree analysis were performed using the tools available in the PubMLST//Chlamydiales database (https://pubmlst.org/ (accessed on 21 January 2022)). Esri ArcGis Desktop 10.6.1 (www.esri.com (accessed on 10 February 2022)) was used for the cartographical analysis.

Multiple-sequence alignment tool Multalin (http://multalin.toulouse.inra.fr/multalin/ (accessed on 21 January 2022)) was used to compare the concatenated sequences of the housekeeping genes for CT strains.

For treatment of chlamydial infections, oral doxycycline 100 mg twice daily over 7 days or alternatively oral azithromycin 1.5 g as a single dose was used in all CT-positive patients.

## 3. Results and Discussion

Overall, we investigated clinical specimens obtained during 2017–2018 from heterosexual chlamydia patients who attended outpatient clinics located in major regions of the Republic of Belarus, such as Brest, Gomel, Grodno, Minsk, Mogilev, Vitebsk and Minsk City (Figure 1). In fact, only 63/1098 (5.7%) of patients tested were CT-positive according to PCR (Table S1). Among them, 31/63 (49.2%) chlamydia patients were asymptomatic and 32/63 (51.8%) had symptoms of typical complaints for genital chlamydial infection [33]. We applied the MLST scheme based on seven housekeeping genes, which were described earlier and successfully used by us to discriminate CT [3,4]. For each DNA sample isolated from individual clinical specimen, the alleles of the seven loci were compared with each other and with the alleles from the PubMLST database for chlamydiales (http://pubmlst.org/chlamydiales/ (accessed on 21 January 2022)) to identify the relevant specific STs. We detected 12 different STs of two types: (i) ST4, ST6, ST9, ST13, ST38, ST95, and ST110 which were already known worldwide; and (ii) novel ST271-275 detected by us in six patients (9.5% from PCR-positive individuals) here for the first time (Table S2). In fact, 5/6 patients (83.3%) were females 21–34 years old, among which 4/5 (80%) demonstrated no symptoms, being asymptomatic similarly to the male patient with ST 271 (Table S2). The greatest diversity of CT strains was seen in two regions, namely in Brest and the combined Minsk City and the Minsk region, in which we detected 9/12 (75%) and 7/12 (58.3%) different STs, respectively (Figure 1 and Figure S1).

In fact, ST4 was predominant (27/63, 42.9%), while two other STs, ST9 and ST13, were found regularly (11/63, 17.5% and 10/63, 15.9%, respectively). In contrast, the rest of STs could be rarely detected and were distributed as follow: ST6 (4/63, 6.4%), ST38 (3/63, 4.8%), ST271 (2/63, 3.2%). ST95 (1/63, 1.6%), ST110 (1/63, 1.6%), ST272 (1/63, 1.6%), ST273 (1/63, 1.6%), ST274 (1/63, 1.6%), and ST275 (1/63, 1.6%). The novel STs were registered in only two regions, namely Minsk (ST271 and ST275) and Brest (ST271, ST272, ST273, and ST274) (Figure 1).

**Figure 2:**
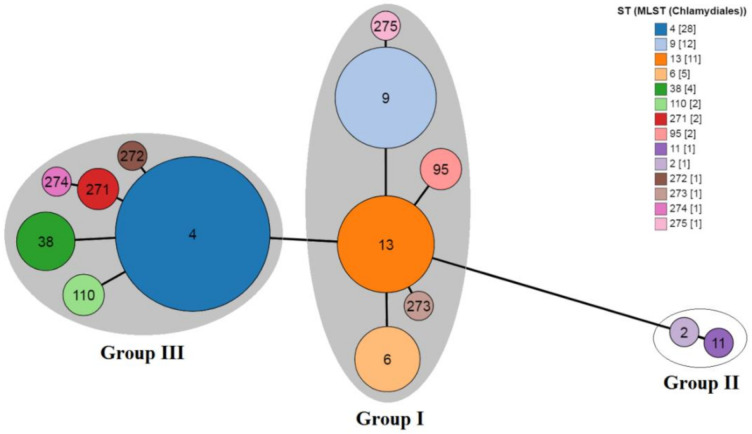
GrapeTree clustering of 14 STs available in the *Chlamydiales* PubMLST database (https://pubmlst.org/chlamydiales/ (accessed on 21 January 2022)). Each node corresponds to a single ST marked with an individual color. The numbers in square brackets indicate *C. trachomatis* representatives in the MLST database. MLST STs of the Group I and the Group III of *C. trachomatis* strains found in our study were marked with gray ovals. The representative STs, ST2 and ST11 of the Group II which are not found in this study were placed in a white oval. The novel STs, ST273 and ST275, belonged to the Group I with ST13 as the founder, and ST271, ST272 and ST274 belonged to the Group III with ST4 as the founder.

The GrapeTree analysis clustered the Belarus CT strains with those obtained from the PubMLST/Chlamydiales database (https://pubmlst.org/ (accessed on 21 January 2022)) into two different groups, namely Group I and Group III (Figure 2) of three Groups I-III assigned earlier by Pannekoek et al. [3] and associated with urogenital strains only.

**Figure 3:**
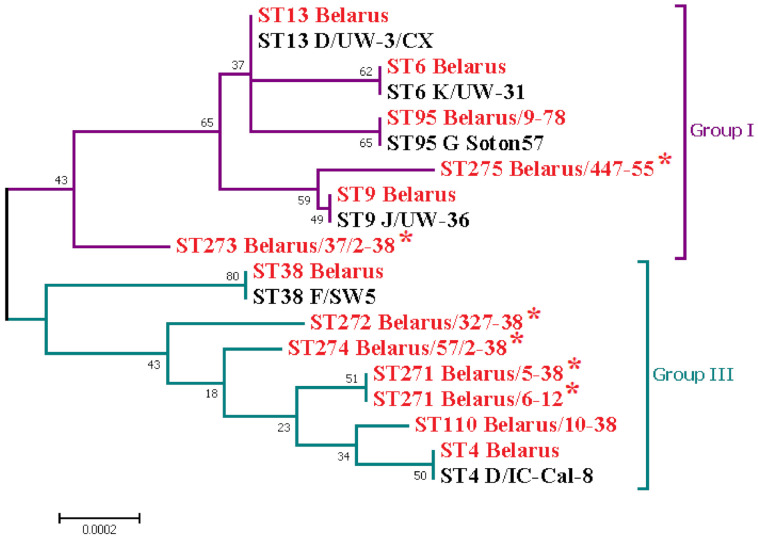
Phylogenetic analysis of concatenated sequences of 7 housekeeping gene fragments of the *C. trachomatis* strains of the ST4, ST6, ST9, ST13, ST38, ST95, ST110, ST271, ST272, ST273, ST274 and ST275 detected in the Republic of Belarus and the representative reference strains with the relevant STs which are available in *Chlamydiales* PubMLST database (https://pubmlst.org/chlamydiales/ (accessed on 21 January 2022)). The evolutionary tree was inferred in MEGA 7.0 using the Neighbor-Joining method with 100 bootstrap replicate samples (the Tamura 3-parameter method [32]). MLST STs of *C. trachomatis* strains found in our study marked with red color. The novel STs 271-275 are marked with a red asterisk.

The first complex consisted of ST6/ST9/ST13/ST95/ST273 and ST275 (ST13 as a founder) and the second one included ST4/ST38/ST110/ST271/ST272 and ST274 (ST4 as a founder). However, in contrast with a neighboring country [3,4], we identified no CT strains belonging to Group II which were found to be associated with LGV strains [3]. Phylogenetic analysis also demonstrated the clustering of the majority of Belarus CT strains with other international CT isolates (Figure 3).

Surprisingly, all six CT strains of five novel STs formed individual branches, suggesting the emergence of the novel evolutionary clonal or subclonal lineages derived from the progenitor STs, such as ST4 (ST271, ST272 and ST274), ST9 (ST275), and ST13 (ST273) (Figure 3). We found only single changes in the *gidA* for ST271, in the *oppA* for ST272 and for ST273, and in both these alleles for ST274 (Table S2) in comparison with the relevant ancestors ST4 and ST13. In contrast, the allele profiles for ST275 and the ancestor ST9 were identical. Comparison of the concatenated sequences of the housekeeping genes for both ST275 and ST9 showed the presence of the SNP in position 126 with the change G→A in ST275 (the CT Belarus/447-55 strain) versus ST9 for both reference CT strain J/UW-36 (ST9) (ID No. 17 in PubMLST) and the CT Belarus/50-38 strain detected in the Brest region of the Republic of Belarus in 2018 (Figure S2).

Overall, our results showed for the first time the genetic diversity of the CT strains found in the Republic of Belarus. We demonstrated the prevalence of ST4 in the majority of the Regions (6/7, 85.7%) of this country. In two of seven regions (28.6%), we report the emergence of five novel CT STs, ST271-275, unique to the world. Recently, no correlation was reported between MLST profiles and symptomatology [34,35]. Importantly, in our research, all six patients were asymptomatic with no reason for medical attention and were identified only as a result of annual routine screening studies. These findings highlight the importance of national screening programs for the early detection of asymptomatic chlamydia patients to prevent the development of chronic forms and further spread of chlamydial infection. The certain limitation of our research can be explained by the predominance of the number of female patients (56/63, 88.9%) compared to the number of examined male ones (7/63, 11.1%). However, this is the first preliminary study that included only random group of heterosexual patients from all regions of the Republic Belarus without any special selection. Based on the data obtained, we intend to significantly expand the survey population in the future investigation in order to obtain more accurate data on the distribution of CT STs in the same regions. The data obtained in this study can contribute to understanding the molecular epidemiology of chlamydia and the formation of the predominant clonal lineages of CT strains worldwide.

## Data Availability

All the data presented are available in PubMLST database (https://pubmlst.org/ (accessed on 21 January 2022), Acc. No. 4464–4526).

## Supplementary Materials

The following are available online at https://www.mdpi.com/article/10.3390/microorganisms10020478/s1, Table S1: Number of CT-positive patients among random cohort of patients from different regions of the Republic of Belarus tested in PCR, Table S2: List of the *C. trachomatis* strains available in PubMLST database and used in this study, Figure S1: The number of STs identified with MLST in seven main regions of the Republic of Belarus in 2017–2018, Figure S2: Multiple sequence alignment of the concatenate seven housekeeping genes such as: *gatA*, *oppA*, *hflX*, *gidA*, *enoA*, *hemN* and *fumC* (Table S2) derived from the CT strains detected in the Republic of Belarus (Belarus/50-38 (ST9), Belarus/447-55 (ST275) and the reference CT strain J/UW-36 (ST9) available in the PubMLST database (https://pubmlst.org/ (accessed on 21 January 2022)) and presented in Table S2. References [36,37,38] have been cited in the Supplementary Materials.
