# Supplementary material for: Emergence of Novel Chlamydia trachomatis Sequence Types among Chlamydia Patients in the Republic of Belarus
Source: Microorganisms. 2022 Feb 21;10(2):478. doi: 10.3390/microorganisms10020478 (PMC8876968; doi:10.3390/microorganisms10020478)
Supplement: Supplementary file 1 [file microorganisms-10-00478-s001.zip › microorganisms-1586044-supplementary.pdf]

Supplemental Table S1. Number of CT-positive patients among random cohort of patients from different Regions of the Republic of Belarus tested in PCR.

**Region**	**Number of patients tested (n)**						**Number of CT-positive patients in PCR* (n)**					
	**Total**		**Male**		**Female**		**Total**		**Male**		**Female**	
	**Abs.**	**%**	**Abs.**	**%**	**Abs.**	**%**	**Abs.**	**%**	**Abs.**	**%**	**Abs.**	**%**
Brest Region	340	30,9	74	21,8	266	78,2	29	8,5	2	2,7	27	10,4
Minsk Region	210	19,1	49	23,3	161	76,7	10	4,8	1	2,1	9	5,6
Mogilev Region	95	8,7	22	23,2	73	76,8	6	6,3	0	0	6	8,2
Grodno Region	76	6,9	21	27,6	55	72,4	5	6,6	3	14,3	2	3,6
Vitebsk Region	94	8,6	23	24,5	71	75,5	5	5,3	1	4,3	4	5,6
Gomel Region	136	12,4	28	20,6	108	79,4	3	2,2	0	0	3	2,8
Minsk City	147	13,4	34	23,1	113	76,9	5	3,4	0	0	5	4,4
Total	1098	100	251	22,9	847	77,1	63	5,7	7	2,8	56	6,6

* AmpliSens® Chlamydia trachomatis-FRT PCR kits (Central Research Institute of Epidemiology, Moscow, Russia) were used for real-time PCR with DNA isolated from clinical specimens of chlamydia patients as described [4].

Supplemental Table S2. List of the *C. trachomatis* strains available in PubMLST database and used in this study.

**Id**	**Isolate**	**Country**	**Region**	**Year**	**Gender**	**Age**	**Manifestations****	**ST**	***gatA***	***oppA***	***hflX***	***gidA***	***enoA***	***hemN***	***fumC***	**Reference / Source**
4464	Belarus/5-38	Republic of Belarus	Brest Region	2018	female	34	**-**	**271**	3	1	1	5	4	2	3	This study
4465	Belarus/6-12	Republic of Belarus	Minsk Region	2017	male	41	**-**	**271**	3	1	1	5	4	2	3	This study
4466	Belarus/327-38	Republic of Belarus	Brest Region	2018	female	31	**-**	**272**	3	3	1	2	4	2	3	This study
4467	Belarus/37/2-38	Republic of Belarus	Brest Region	2018	female	21	**-**	**273**	3	1	2	5	3	2	3	This study
4468	Belarus/57/2-38	Republic of Belarus	Brest Region	2018	female	25	**-**	**274**	3	4	1	5	4	2	3	This study
446	Belarus/447-55	Republic of	Minsk Region	201	female	28	**+**	**27**	3	3	2	4	4	2	3	This

9		Belarus		8				**5**								study
4470	Belarus/3-20	Republic of Belarus	Mogilev Region	2017	female	31	+	**38**	3	1	2	2	4	2	3	This study
4471	Belarus/5-44	Republic of Belarus	Vitebsk Region	2017	male	24	+	**38**	3	1	2	2	4	2	3	This study
4472	Belarus/23-38	Republic of Belarus	Brest Region	2018	female	27	-	**38**	3	1	2	2	4	2	3	This study
4473	Belarus/17-40	Republic of Belarus	Mogilev Region	2018	female	27	+	**4**	3	1	1	2	4	2	3	This study
4474	Belarus/257-38	Republic of Belarus	Brest Region	2018	male	20	+	**4**	3	1	1	2	4	2	3	This study
4475	Belarus/67-38	Republic of Belarus	Brest Region	2017	female	22	+	**4**	3	1	1	2	4	2	3	This study
4476	Belarus/586-75	Republic of Belarus	Minsk Region	2018	female	22	-	**4**	3	1	1	2	4	2	3	This study
4477	Belarus/8936-48	Republic of Belarus	Mogilev Region	2018	female	34	+	**4**	3	1	1	2	4	2	3	This study
4478	Belarus/4-78	Republic of Belarus	Minsk City	2018	female	21	+	**4**	3	1	1	2	4	2	3	This study
4479	Belarus/8-38	Republic of Belarus	Brest Region	2018	female	27	-	**4**	3	1	1	2	4	2	3	This study
4480	Belarus/46/1-38	Republic of Belarus	Brest Region	2018	female	35	-	**4**	3	1	1	2	4	2	3	This study
4481	Belarus/13/1-38	Republic of Belarus	Brest Region	2018	female	27	-	**4**	3	1	1	2	4	2	3	This study
4482	Belarus/191-38	Republic of Belarus	Brest Region	2018	male	N/A*	+	**4**	3	1	1	2	4	2	3	This study
4483	Belarus/4-38	Republic of Belarus	Brest Region	2018	female	20	+	**4**	3	1	1	2	4	2	3	This study
4484	Belarus/4/2-38	Republic of Belarus	Brest Region	2017	female	29	-	**4**	3	1	1	2	4	2	3	This study
4485	Belarus/14-38	Republic of Belarus	Brest Region	2018	female	21	+	**4**	3	1	1	2	4	2	3	This study

4486	Belarus/45/1-38	Republic of Belarus	Brest Region	2018	female	24	-	**4**	3	1	1	2	4	2	3	This study
4487	Belarus/9983-58	Republic of Belarus	Mogilev Region	2018	female	25	+	**4**	3	1	1	2	4	2	3	This study
4488	Belarus/53-37	Republic of Belarus	Minsk Region	2018	female	22	-	**4**	3	1	1	2	4	2	3	This study
4489	Belarus/7-12	Republic of Belarus	Minsk Region	2017	female	23	+	**4**	3	1	1	2	4	2	3	This study
4490	Belarus/11-72	Republic of Belarus	Vitebsk Region	2018	female	33	+	**4**	3	1	1	2	4	2	3	This study
4491	Belarus/13-72	Republic of Belarus	Vitebsk Region	2018	female	42	+	**4**	3	1	1	2	4	2	3	This study
4492	Belarus/14-72	Republic of Belarus	Vitebsk Region	2018	female	22	+	**4**	3	1	1	2	4	2	3	This study
4493	Belarus/7-80	Republic of Belarus	Vitebsk Region	2018	female	20	+	**4**	3	1	1	2	4	2	3	This study
4494	Belarus/3271	Republic of Belarus	Grodno Region	2018	male	34	+	**4**	3	1	1	2	4	2	3	This study
4495	Belarus/626-75	Republic of Belarus	Minsk Region	2018	female	23	-	**4**	3	1	1	2	4	2	3	This study
4496	Belarus/155-68	Republic of Belarus	Minsk Region	2018	female	22	-	**4**	3	1	1	2	4	2	3	This study
4497	Belarus/8-88	Republic of Belarus	Mogilev Region	2018	female	29	-	**4**	3	1	1	2	4	2	3	This study
4498	Belarus/347-38	Republic of Belarus	Brest Region	2018	female	39	-	**4**	3	1	1	2	4	2	3	This study
4499	Belarus/8-38/2	Republic of Belarus	Brest Region	2018	female	22	-	**4**	3	1	1	2	4	2	3	This study
4500	Belarus/154-38	Republic of Belarus	Brest Region	2017	female	20	+	**13**	3	3	2	5	3	2	3	This study
4501	Belarus/39799	Republic of Belarus	Gomel Region	2017	female	18	-	**13**	3	3	2	5	3	2	3	This study
450	Belarus/3-38	Republic of	Brest Region	201	female	18	+	**13**	3	3	2	5	3	2	3	This

2		Belarus		8													study
4503	Belarus/54/1-38	Republic of Belarus	Brest Region	2018	female	24	-	**13**	3	3	2	5	3	2	3		This study
4504	Belarus/98-38	Republic of Belarus	Brest Region	2018	female	28	-	**13**	3	3	2	5	3	2	3		This study
4505	Belarus/9-88	Republic of Belarus	Minsk city	2018	female	21	+	**13**	3	3	2	5	3	2	3		This study
4506	Belarus/1268	Republic of Belarus	Minsk Region	2017	female	23	-	**13**	3	3	2	5	3	2	3		This study
4507	Belarus/160-38	Republic of Belarus	Brest Region	2018	female	22	+	**13**	3	3	2	5	3	2	3		This study
4508	Belarus/37-38	Republic of Belarus	Brest Region	2018	female	19	-	**13**	3	3	2	5	3	2	3		This study
4509	Belarus/449	Republic of Belarus	Minsk Region	2017	female	23	+	**13**	3	3	2	5	3	2	3		This study
4510	Belarus/14-10	Republic of Belarus	Minsk city	2017	female	29	+	**9**	3	3	2	4	3	2	3		This study
4511	Belarus/6-28	Republic of Belarus	Grodno Region	2018	female	24	+	**9**	3	3	2	4	3	2	3		This study
4512	Belarus/5-61	Republic of Belarus	Grodno Region	2018	female	24	+	**9**	3	3	2	4	3	2	3		This study
4513	Belarus/7-38	Republic of Belarus	Brest Region	2018	female	25	-	**9**	3	3	2	4	3	2	3		This study
4514	Belarus/50-38	Republic of Belarus	Brest Region	2018	female	25	-	**9**	3	3	2	4	3	2	3		This study
4515	Belarus/43/2-38	Republic of Belarus	Brest Region	2018	female	24	-	**9**	3	3	2	4	3	2	3		This study
4516	Belarus/1/2-38	Republic of Belarus	Brest Region	2017	female	40	-	**9**	3	3	2	4	3	2	3		This study
4517	Belarus/5-29	Republic of Belarus	Grodno Region	2018	male	23	+	**9**	3	3	2	4	3	2	3		This study
4518	Belarus/40-38	Republic of Belarus	Brest Region	2018	female	29	-	**9**	3	3	2	4	3	2	3		This study

4519	Belarus/8-12	Republic of Belarus	Minsk Region	2017	female	23	+	**9**	3	3	2	4	3	2	3	This study
4520	Belarus/737	Republic of Belarus	Grodno Region	2018	male	35	+	**9**	3	3	2	4	3	2	3	This study
4521	Belarus/7-20	Republic of Belarus	Mogilev Region	2017	female	27	+	**6**	3	3	2	5	3	1	3	This study
4522	Belarus/1552	Republic of Belarus	Gomel Region	2018	female	24	-	**6**	3	3	2	5	3	1	3	This study
4523	Belarus/4610	Republic of Belarus	Gomel Region	2018	female	24	-	**6**	3	3	2	5	3	1	3	This study
4524	Belarus/14-88	Republic of Belarus	Minsk city	2018	female	22	+	**6**	3	3	2	5	3	1	3	This study
4525	Belarus/9-78	Republic of Belarus	Minsk city	2018	female	30	+	**95**	3	3	28	5	3	2	3	This study
4526	Belarus/10-38	Republic of Belarus	Brest Region	2018	female	20	-	**110**	3	1	1	2	3	2	3	This study
240	F/SW5	Sweden	N/A	2002	female	N/A	N/A	**38**	3	1	2	2	4	2	3	[36]
7	D/IC-Cal-8	N/A	N/A	N/A	N/A	N/A	N/A	**4**	3	1	1	2	4	2	3	[3]
215	D/UW-3/CX	USA	Seattle	1965	female	N/A	N/A	**13**	3	3	2	5	3	2	3	[3, 36, 37]
17	J/UW-36	N/A	N/A	N/A	N/A	N/A	N/A	**9**	3	3	2	4	3	2	3	[3]
14	K/UW-31	USA	Washington	1973	female	N/A	N/A	**6**	3	3	2	5	3	1	3	[3]
567	G_Soton57	UK	N/A	2009	female	N/A	N/A	**95**	3	3	28	5	3	2	3	[38]
646	F_S4410	Sweden	N/A	2010	female	N/A	N/A	**110**	3	1	1	2	3	2	3	[38]

* N/A - Not Available. Changed alleles in the Belarus CT strains in comparison with the relevant ancestors are marked in red. ** - Presence symptoms of typical complaints for genital chlamydial infection [33] or absence (the asymptomatic chlamydia patient).

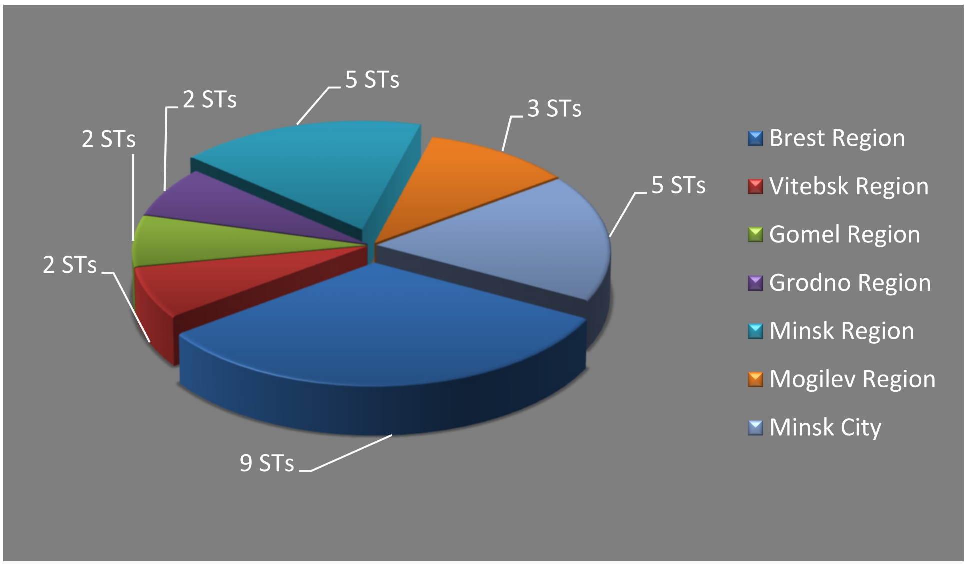

Figure S1. The number of CT STs identified with MLST in seven main Regions of the Republic of Belarus in 2017-2018.

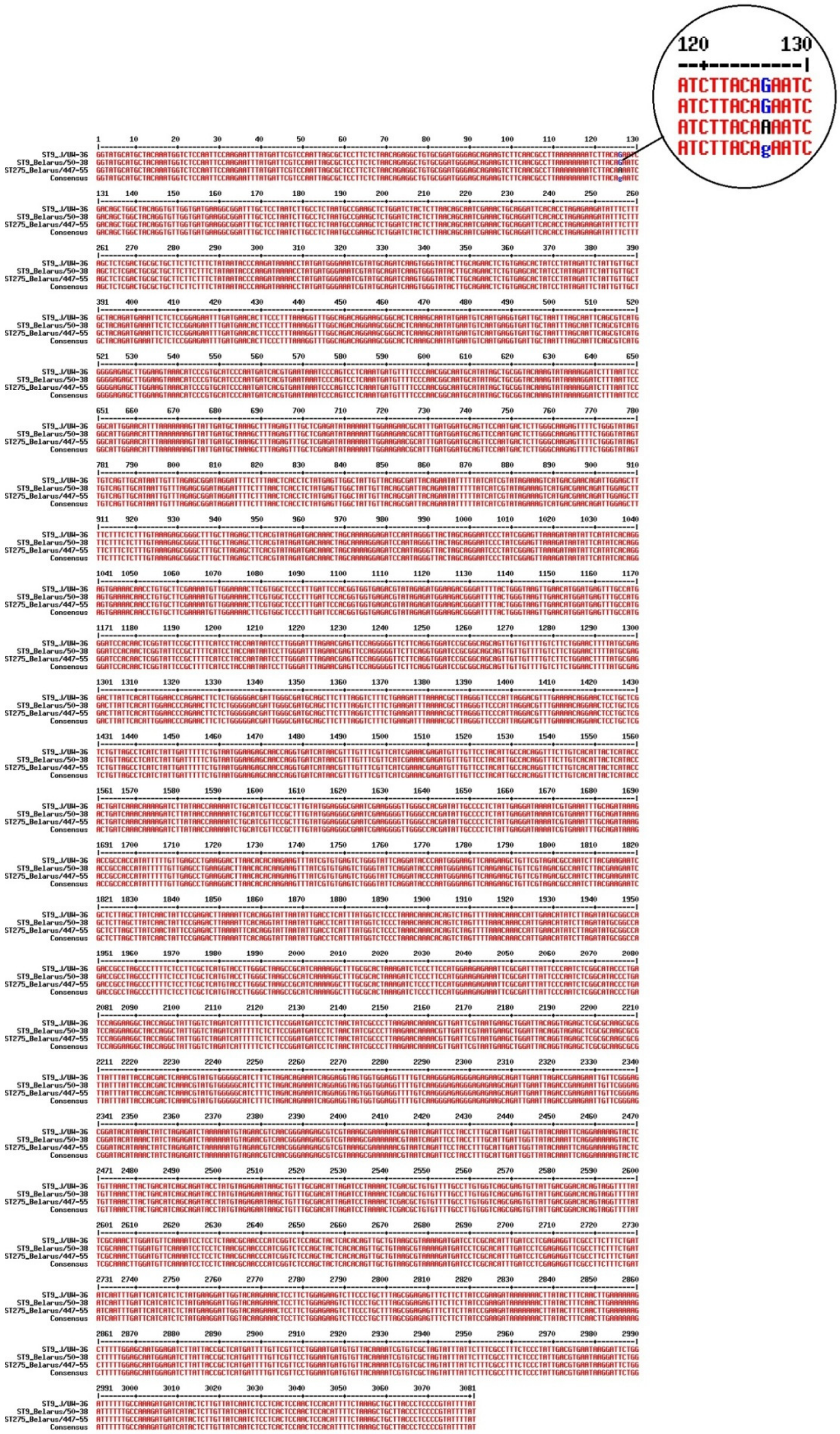

Supplemental Figure S2. Multiple sequence alignment of the concatenate seven housekeeping genes such as: *gatA*, *oppA*, *hflX*, *gidA*, *enoA*, *hemN* and *fumC* (Table S2) derived from the CT strains detected in the Republic of Belarus (Belarus/50-38 (ST9), Belarus/447-55 (ST275) and the reference CT strain J/UW-36 (ST9) available in the PubMLST database (https://pubmlst.org/) and presented in Table S1.
